# Deep Learning Imaging Reconstruction Algorithm for Carotid Dual Energy CT Angiography: Opportunistic Evaluation of Cervical Intervertebral Discs—A Preliminary Study

**DOI:** 10.1007/s10278-024-01016-x

**Published:** 2024-03-01

**Authors:** Chenyu Jiang, Jingxin Zhang, Wenhuan Li, Yali Li, Ming Ni, Dan Jin, Yan Zhang, Liang Jiang, Huishu Yuan

**Affiliations:** 1 Department of Radiology, Peking University Third Hospital, Beijing, China; 2 Department of Integration of Chinese and Western Medicine, School of Basic Medical Sciences, Peking University, Beijin, China; 3 CT Research Center, GE Healthcare China, 1 South Tongji Road, Beijing, China; 4 Department of Orthopaedics, Peking University Third Hospital, Beijing, China

**Keywords:** Intervertebral disc, Dual-energy computed tomography, Deep learning, Image reconstruction

## Abstract

Thus, the aim of this study is to evaluate the performance of deep learning imaging reconstruction (DLIR) algorithm in different image sets derived from carotid dual-energy computed tomography angiography (DECTA) for evaluating cervical intervertebral discs (IVDs) and compare them with those reconstructed using adaptive statistical iterative reconstruction-Veo (ASiR-V). Forty-two patients who underwent carotid DECTA were included in this retrospective analysis. Three types of image sets (70 keV, water-iodine, and water-calcium) were reconstructed using 50% ASiR-V and DLIR at medium and high levels (DLIR-M and DLIR-H). The diagnostic acceptability and conspicuity of IVDs were assessed using a 5-point scale. Hounsfield Units (HU) and water concentration (WC) values of the IVDs; standard deviation (SD); and coefficient of variation (CV) were calculated. Measurement parameters of the 50% ASIR-V, DLIR-M, and DLIR-H groups were compared. The DLIR-H group showed higher scores for diagnostic acceptability and conspicuity, as well as lower SD values for HU and WC than the ASiR-V and DLIR-M groups for the 70 keV and water-iodine image sets (all *p* < .001). However, there was no significant difference in scores and SD among the three groups for the water-calcium image set (all *p* > .005). The water-calcium image set showed better diagnostic accuracy for evaluating IVDs compared to the other image sets. The inter-rater agreement using ASiR-V, DLIR-M, and DLIR-H was good for the 70 keV image set, excellent for the water-iodine and water-calcium image sets. DLIR improved the visualization of IVDs in the 70 keV and water-iodine image sets. However, its improvement on color-coded water-calcium image set was limited.

## Introduction

Carotid computed tomography angiography (CTA) is an effective imaging technique for detecting carotid artery pathology [1]. It has become the fastest growing imaging modality in the emergency department for patients suspected of cerebrovascular disease [2]. In clinical setting, it is important to assess both the carotid arteries and the cervical intervertebral discs (IVDs). There are overlapping symptoms between intervertebral disc lesions and vascular lesions, such as headache, vertigo, and limb sensory motor disorder [3–5]. Therefore, it would be advantageous to evaluate both the cervical IVDs and the carotid arteries using a single imaging modality. However, conventional CT technology is not suitable for evaluating IVDs due to its low soft tissue resolution and susceptibility to beam hardening artifacts [6, 7].

Recent studies have shown that different dual-energy CT (DECT) image sets derived from virtual monochromatic image (VMI) and material decomposition (MD) techniques had different potential to eliminate beam hardening and improve the contrast resolution for evaluating IVDs [8–11]. But no studies have systematically compared the diagnostic performance of VMI and different water-based MD image sets for IVD herniation. Additionally, several studies have shown improved perceptual image quality and standard metrics (e.g., contrast-to-noise ratio) using deep learning imaging reconstruction (DLIR) when compared with filtered back projection and iterative reconstruction [12–15]. However, DLIR has not been thoroughly evaluated for its ability to improve IVD display, especially on the water-based MD image sets. We hypothesized that the DLIR algorithm applied for carotid dual-energy computed tomography angiography (DECTA) would improve the display of IVDs.

Thus, the aim of this study was to evaluate the performance of DLIR in DECTA for opportunistic evaluation of IVDs. This involved evaluating the effectiveness of DLIR for three types of image sets: 70 keV, water (iodine), and color-coded water (calcium) images or “virtual noncalcium (VNCa)” image sets, in comparison with adaptive statistical iterative reconstruction-Veo at a level of 50% (ASiR-V).

## Materials and Methods

### Study Population

This study is a retrospective analysis of data from a prior prospective study conducted between March 2023 and April 2020. The original prospective study had received approval from the institutional review board, and all participants had given informed consent. The present retrospective investigation was also approved by the institutional review board, with no need for additional consent. A total of 63 consecutive patients who were part of the earlier prospective study between March 2023 and April 2020 were included in this analysis. Among them, 42 patients were enrolled for final evaluation of cervical intervertebral discs. Patients with spinal malignancy (*n* = 1), severe spondylodiscitis (*n* = 13), or those who had undergone dorsal instrumentation or intervertebral spacer placement (*n* = 7) were excluded.

### Carotid DECTA Protocol

All examinations were performed by a fast kilovoltage switching CT scanner (revolution CT; GE Healthcare). The scan protocols for DECT were as follows: 80/140 kV peak tube voltage, variable tube current (GSI Assist; GE Healthcare), 12 HU noise index at 5-mm section collimation 128 detectors with 0.625-mm section thickness, 80 mm beam collimation, 0.5-s rotation time, 0.984:1 pitch, 36 cm display field of view. The nonionic-iodinated contrast agent (370 mgI/mL, Omnipaque 350, GE Healthcare, Shanghai, China) was injected intravenously into the right cubital at a rate of 3.5 mL/s using an automatic injector with a bolus of 40 mL and followed by 30 mL saline flush at the same injection rate. The bolus-tracking technique was used with a trigger threshold of 120 HU in the ascending aorta. CT data acquisition started 5 s after triggering, scanning from the cranial crest to the aortic arch in the craniocaudal direction.

### Imaging Reconstruction and Postprocessing

All images were reconstructed with the algorithm of adaptive statistical iterative reconstruction-Veo at a level of 50% (ASiR-V50%, GE Healthcare) and deep-learning image reconstruction (DLIR, GE Healthcare) at medium (DLIR-M) and high (DLIR-H) levels. All CT images were reconstructed with the standard reconstruction kernel and a slice thickness of 0.625 mm.

**Fig. 1:**
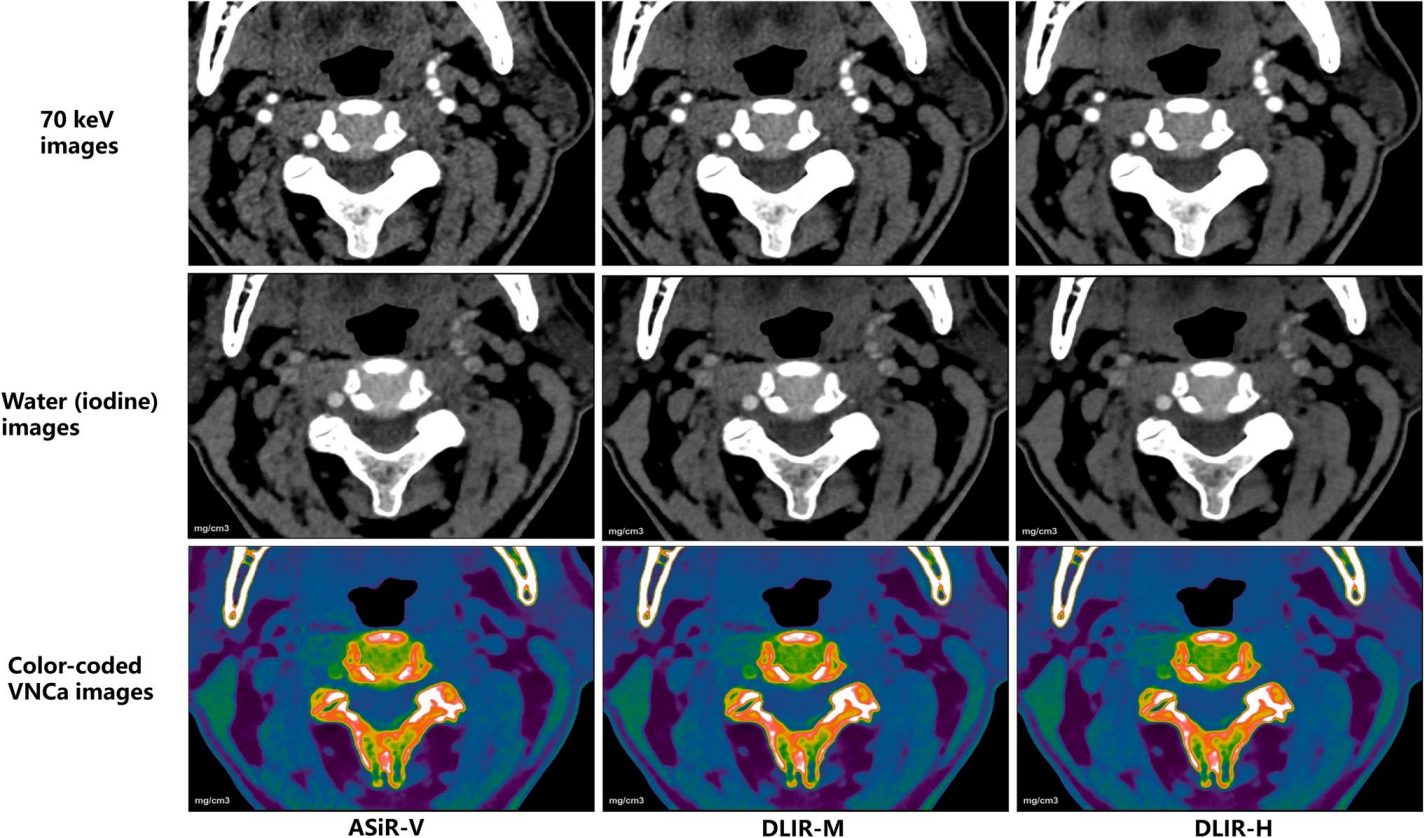
Axial CT images of a 66-year-old man with central disc herniation at the C2/3 level. The 70 keV, water (iodine), and color-coded virtual noncalcium (VNCa) images were reconstructed using iterative reconstruction-Veo (ASiR-V), deep learning image reconstruction at medium level (DLIR-M), and high level (DLIR-H). The deep learning image reconstruction algorithms effectively reduced image noise of grayscale CT images compared to ASiR-V

VMI at 70 keV, water (iodine), and color-coded water (calcium) image sets with ASiR-V50%, DLIR-M, and DLIR-H (Fig. 1) were generated using GSI Viewer software (Advantage Workstation, version 4.7, GE Healthcare).

### Quantitative Image Analysis

The CT number (HU) and image noise values (SD) of each IVD on 70 keV images were measured by a radiologist (C.J. with 5 years’ experience of interpreting CT images). To avoid a partial volume effect of the adjacent bone or cervical spinal cord, ROIs were placed in the central region of each IVD. The signal-to-noise ratio (SNR) and contrast-to-noise ratio (CNR) of 70 keV images were calculated as: SNR = disk HU / disk SD, CNR = (disc HU – spinal cord HU)/ spinal cord SD. Then the ROIs were cloned to the water (iodine) and water (calcium) image sets to measure the water concentration (WC) and SD of each IVD. To assess the variability in measured values, we calculated the coefficient of variation (CV) by dividing the SD by the mean WC of each IVD.

### Qualitative Image Analysis

Two experienced radiologist (C.J. and W.L. with 5 and 10 years of post-training experience in interpreting musculoskeletal CT images, respectively), who were blinded to clinical and CT reconstruction information, assessed the conspicuity of IVDs and diagnostic confidence using 70 keV, water (iodine), and color-coded water (calcium) image sets reconstructed by ASiR-V, DLIR-M, and DLIR-H algorithms. The diagnostic acceptability and conspicuity of IVDs were qualitatively assessed using a 5-point scale, with a range from 1 to 5 (1 = poor opacification of the IVD or unacceptable for diagnostic; 5 = excellent opacification of the IVD).

The diagnosis reference for each IVD was determined by a consensus between a senior orthopedical surgeon (with 20 years of spine orthopedical experience) and a senior radiologist (with 20 years of experience in interpreting musculoskeletal images). This determination was based on the analysis of three image sets by two reviewers who were unaware of clinical data and reconstruction information in CT images, in a randomized order for algorithms. The classification of abnormalities in IVDs was according to the lumbar disk pathologic classification of the North American Spine Society [16]. The cervical disks were classified as abnormal or normal. Abnormal disks included herniation (protrusion or extrusion with focal displacement of disk material < 25% of the disk circumference) and bulging disks (disk tissue extending beyond the edges of the disk space > 25% of the disk circumference). Window setting were adjusted during qualitative assessment. There was an interval of 4 weeks between the evaluation of ASiR-V and DLIR images to avoid potential recall bias.

### Statistical Analysis

All statistical analyses were performed using SPSS (Statistics version 26.0 for Windows, IBM). The Kolmogorov–Smirnov test was performed to assess the normality of data before further statistical analyses. The differences in SNR, CNR, WC, SD, and CV among 70 keV, water (iodine) and water (calcium) image sets reconstructed with ASiR-V 50%, DLIR-M and DLIR-H were compared using repeated measure ANOVA with the Bonferroni post hoc test. The Friedman test was used to compared the scores among ASiR-V, DLIR-M, and DLIR-H algorithms in three image sets. Sensitivities, specificities, positive predictive values (PPVs), negative predictive value (NPVs), and accuracy values were calculated on a per-disk basis. Inter-rater agreements for evaluating IVDs were tested using Cohen’s kappa test, using the following criteria: poor (*κ* < 0.4); moderate (*κ* = 0.41–0.60); good (*κ* = 0.61–0.80); excellent (*κ* = 0.81–1.00) and differences evaluated using the McNemar-Bowker test. Power analysis was performed with a repeated measures ANOVA design. A total of 40 patients achieves 80% power if a Geisser–Greenhouse corrected *F* test is used with a 5% significance level.

## Results

### Participants’ Information

A total of 42 participants with 210 cervical intervertebral discs were included in the study. Out of these, 197 discs were finally analyzed while 13 were excluded due to sclerosis artifacts (*n* = 5) and severe narrowing of the disc space (*n* = 8). The participants’ demographics and clinical information are shown in Table 1.

**Table 1:** Characteristics of the participants (*n* = 42)

Characteristic	Value
Overall age (yeas)	56.5 ± 14.0
Overall BMI (kg/m^2^)	24.9 ± 3.3
Women	21/42 (50%)
Age (yeas)	54.3 ± 12.1
BMI (kg/m^2^)	25.1 ± 3.8
Men	21/42 (50%)
Age (yeas)	58.9 ± 15.6
BMI (kg/m^2^)	24.7 ± 2.9
Known vascular disease	
Atherosclerosis	22 (52.4%)
Carotid body tumor	7 (16.7%)
Intracranial aneurysm	2 (4.7%)
Vascular malformation	2 (4.7%))
No. of cervical spondylosis	9 (21.4%)

Data given are mean ± standard deviation or absolute numbers (percentage).

*BMI* body mass index

### Quantitative Image Analysis

DLIR-M and DLIR-H significantly improved the SNR and CNR of IVDs in 70 keV images compared with ASiR-V (all *p* < 0.001) (Table 2). The SNR values for ASiR-V, DLIR-M, and DLIR-H were 5.73 ± 2.5, 6.93 ± 2.8, and 8.43 ± 3.3, respectively, while the CNR values for ASiR-V, DLIR-M, and DLIR-H were 3.86 ± 1.2, 4.75 ± 1.8, and 5.78 ± 1.9, respectively.

**Table 2:** Quantitative image analysis among three reconstruction image sets

	ASiR-V	DLIR-M	DLIR-H	*p*-value
70 keV image				
HU	106.58 ± 20.1	103.79 ± 18.5	103.88 ± 1.3	0.256
SD	19.17 ± 7.2	17.43 ± 7.3	15.97 ± 7.3^⁎^	< 0.001
SNR	5.73 ± 2.5	6.93 ± 2.8^⁎^	8.43 ± 3.3^⁎a^	< 0.001
CNR	3.86 ± 1.2	4.75 ± 1.8^⁎^	5.78 ± 1.9^⁎a^	< 0.001
Water (iodine) image				
WC	1080.69 ± 14.5	1077.68 ± 14.6	1077.54 ± 14.8	0.056
SD	8.39 ± 2.5	7.62 ± 2.5^⁎^	6.9 ± 2.4^⁎a^	< 0.001
CV	0.00843 ± 0.0070	0.00707 ± 0.0023^⁎^	0.00635 ± 0.0020^⁎^	< 0.001
VNC image				
WC	1073.17 ± 14.9	1070.36 ± 15.5	1070.22 ± 15.51	0.100
SD	6.35 ± 2.0	6.16 ± 2.1	5.87 ± 2.1	0.072
CV	0.00592 ± 0.0018	0.00578 ± 0.0020	0.00548 ± 0.020	0.083

Data given are mean ± standard deviation

*ASiR-V* adaptive statistical iterative reconstruction-Veo, *DLIR-M* deep learning image reconstruction at medium strength, *DLIR-H* deep learning image reconstruction at high strength, *HU* Hounsfield units, *SD* image noise values, *SNR* signal-to-noise ratio, *CNR* contrast-to-noise ratio, *WC* water concentration, *CV* coefficient of variation, *VNCa* virtual noncalcium

^⁎^Value was statistically different from those with ASiR-V groups

^a^Value was statistically different from those with DLIR-M groups

In water (iodine) image set, there was no significant difference among the ASiR-V, DLIR-M, and DLIR-H algorithm (all *p* > 0.05). However, the DLIR algorithm showed significantly lower SD and CV for WC compared with ASiR-V (all *p* < 0.001) (Table 2). Specifically, the SD values for ASiR-V, DLIR-M, and DLIR-H were 8.39 ± 2.5, 7.62 ± 2.5, and 6.9 ± 2.4, respectively, while the CV values for ASiR-V, DLIR-M, and DLIR-H were 0.00843 ± 0.0070, 0.00707 ± 0.0023, and 0.00635 ± 0.0020, respectively.

In water (calcium) or VNCa image set, the DLIR-H algorithm exhibited the lowest SD and CV. However, there was no statistically significant difference in SD and CV among DLIR-H, DLIR-M, and ASiR-V algorithms (all *p* > 0.05).

### Qualitative Image Analysis

The subjective score for diagnostic acceptability and conspicuity of IVDs of ASiR-V, DLIR-M, and DLIR-H image sets rated by two readers was shown in Table 3. Regardless of 70 keV images, water(iodine) images, or VNCa images, good to excellent interobserver agreement were noted (Table 3). Moreover, the interobserver agreement was highest in the reconstructed image set based on DLIR-H (0.74 for 70 keV; 0.76 for water (iodine); 0.83 for VNCa images).

**Table 3:** Subjective ratings for qualitative image analysis by two reviewers

	Reviewer 1	Reviewer2	*κ*-value	*p*-value
ASiR-V				
70 keV image	2.67 ± 0.76	2.73 ± 0.75	0.71	0.000
Water(iodine)	3.41 ± 0.81	3.56 ± 0.81	0.67	0.000
VNCa	3.93 ± 0.73	3.84 ± 0.74	0.69	0.000
DLIR-M				
70 keV	2.82 ± 0.77	2.89 ± 0.81	0.68	0.000
Water(iodine)	3.54 ± 0.82	3.48 ± 0.83	0.67	0.000
VNCa	3.92 ± 0.73	3.83 ± 0.73	0.73	0.000
DLIR-H				
70 keV	3.01 ± 0.82	3.15 ± 0.81	0.74	0.000
Water(iodine)	3.91 ± 0.91	3.73 ± 0.87	0.76	0.000
VNCa	3.99 ± 0.75	3.92 ± 0.73	0.83	0.000

*ASiR-V* adaptive statistical iterative reconstruction-Veo, *DLIR-M* deep learning image reconstruction at medium strength, *DLIR-H* deep learning image reconstruction at high strength, *VNCa* virtual noncalcium images

*κ*-value and *p*-value were calculated by Cohen’s kappa test

**Fig. 2:**
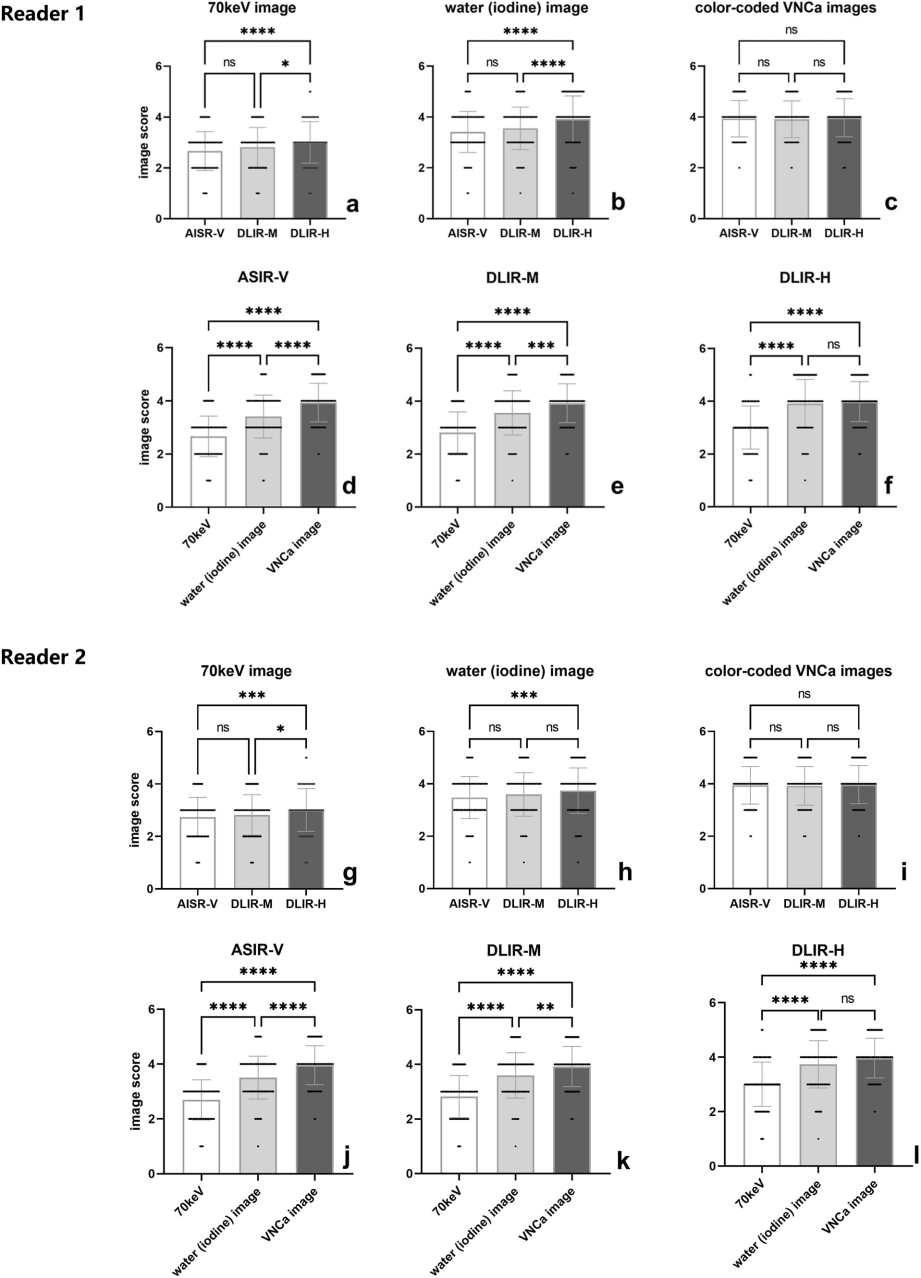
Box and dot plots compared image quality (diagnostic acceptability and conspicuity) of 70 keV (**a**, **g**), water (iodine) (**b**, **h**) and color-coded virtual noncalcium (VNCa) (**c**, **i**) image sets among different reconstruction algorithm, and iterative reconstruction-Veo (ASiR-V) (**d**, **j**), deep learning image reconstruction algorithm at medium level (DLIR-M) (**e**, **k**), at high level (DLIR-H) (**f**, **i**) among different image sets by both readers (**a**–**f** for reader 1; **g**–**i** for reader 2). **** indicates *p* < 0.001, *** indicates *p* < 0.01, and ns indicates means result was not statistical significance

The comparison of qualitative analysis of three reconstruction image sets (ASiR-V, DLIR-M, and DLIR-H) by both readers was shown in Fig. 2. The DLIR algorithm improved the display and diagnostic acceptability of each IVD in both 70 keV (Fig. 1a, g) and water (iodine) image sets (Fig. 1b, h). This improvement was more pronounced as the strength of DLIR algorithm increases. In contrast, there were no significant differences observed for IVD display in color-coded VNCa image set using the three reconstruction algorithms (Fig. 1c, i).

Among three different image sets, the 70 keV image set showed the lowest subjective score. It was followed by the water (iodine) image set, while the color-coded VNCa image set was found to be most favorable for disc evaluation in both ASiR-V and DLIR-M algorithms (Fig. 1d–f, j–i). Using the DLIR-H algorithm, the grayscale water (iodine) image set showed comparable results to the color-coded VNCa image set in evaluating IVDs (Fig. 1f, i).

### Diagnostic Accuracy for IVD Abnormalities per Disk

According to the consensus of two senior radiologists, out of the 197 cervical intervertebral discs (IVDs) evaluated, 77 (39%) were classified as abnormal, while 120 (61%) were classified as normal. The abnormalities observed included bulging in 25 cervical IVDs (12.6%) and herniation in 52 cervical IVDs (26.4%). The diagnostic performance of 70 keV, water (iodine) and VNCa image sets using three reconstruction algorithms for detecting IVD abnormalities is summarized in Table 4. The VNCa image set showed superior diagnostic accuracy, sensitivity, specificity, positive predictive value, and negative predictive value compared to the 70 keV and water (iodine) image sets. Among three different algorithms used for the VNCa image set, DLIR-M demonstrated higher diagnostic accuracy than ASiR-V and DLIR-H. However, these differences were not statistically significant (compared to DLIR-H, *p* = 0.446; compared to ASiR-V, *p* = 0.675).

**Table 4:** Diagnostic efficiency of 70 keV images, water (iodine) images, and VNCa images of three reconstruction image sets for IVD herniation per disk

	Sensitivity	Specificity	PPV	NPV	Accuracy	*p*-value
70 keV image						
ASiR-V	86.9% [0.80–0.91]	69.2% [0.50–0.80]	88.7% [0.82–0.92]	65.4% [0.52–0.76]	81.2% [0.76–0.86]	0.092
DLIR-M	86.2% [0.79–0.90]	73.1% [0.59–0.83]	89.9% [0.83–0.93]	65.5% [0.52–0.76]	82.7% [0.77–0.87]	0.375
DLIR-H	87.5% [0.81–0.92]	67.3% [0.53–0.78]	88.1% [0.81–0.92]	66.0% [0.52–0.77]	82.2% [0.76–0.87]	0.463
Water (iodine) image						
ASiR-V	87.6% [0.81–0.92]	75.0% [0.61–0.84]	90.1% [0.84–0.94]	68.4% [0.55–0.79]	84.3% [0.78–0.89]	0.615
DLIR-M	85.5% [0.79–0.90]	73.0% [0.59–0.83]	89.8% [0.83–0.94]	64.4% [0.52–0.75]	82.2% [0.76–0.86]	0.215
DLIR-H	87.6% [0.81–0.92]	71.1% [0.57–0.81]	89.4% [0.83–0.93]	67.2% [0.54–0.78]	83.2% [0.77–0.88]	0.436
VNCa image						
ASiR-V	89.6% [0.83–0.93]	76.9% [0.63–0.86]	91.5% [0.85–0.95]	72.7% [0.59–0.82]	86.3% [0.81–0.90]	0.123
DLIR-M	92.4% [0.86–0.95]	78.8% [0.65–0.87]	92.4% [0.86–0.96]	78.8% [0.65–0.87]	88.8% [0.83–0.92]	0.392
DLIR-H	88.2% [0.82–0.92]	82.6% [0.70–0.90]	93.4% [0.87–0.96]	71.6% [0.59–0.81]	86.8% [0.81–0.91]	0.639

Data in brackets are 95% confidence intervals

*ASiR-V *adaptive statistical iterative reconstruction-Veo, *DLIR-M* deep learning image reconstruction at medium strength, *DLIR-H* deep learning image reconstruction at high strength, *NPV* negative predictive value, *PPV* positive predictive value, *VNCa* virtual noncalcium

*p*-value was calculated by McNemar-Bowker test

The inter-rater agreement for detecting IVD abnormalities using ASiR-V, DLIR-M, and DLIR-H was good (*κ* = 0.71, 0.72, and 0.69, respectively) for the 70 keV image set. It was excellent for the water (iodine) image set (*κ* = 0.91, 0.85, and 0.86, respectively) and the VNCa image set (*κ* = 0.86, 0.89, and 0.85, respectively).

## Discussion

In the present study, we evaluated the performance of DLIR in three types of image sets: 70 keV, water (iodine), and color-coded water (calcium) or “VNCa” images, derived from carotid DECTA for opportunistic evaluation of cervical IVDs, and compare them with those of images reconstructed using ASiR-V. We found that the DLIR-H group showed significantly higher scores for diagnostic acceptability and conspicuity, as well as lower SD values for HU and WC than the ASiR-V and DLIR-M groups for both the 70 keV and water (iodine) image sets (all *p* < 0.001). However, there was no significant difference in scores for acceptability and conspicuity, as well as SD of HU and WC values, among the ASiR-V, DLIR-M, and DLIR-H groups for the VNCa image set. The VNCa image set showed better diagnostic accuracy compared to 70 keV and water (iodine) image sets for evaluating IVDs, on a per intervertebral disk basis.

Conventional grayscale CT showed moderate diagnostic accuracy of for evaluating disk herniation, due to its low soft tissue resolution and susceptibility to beam hardening artifacts [6]. DECT enables the creation of VMI in the range of 40 to 140 keV, along with water-based MD images using material decomposition technique. In our study, the water (iodine) image set showed higher subjective scores compared to the 70 keV image set when using the same reconstruction algorithm. This finding is consistent with recent results by Wu et al. [8]. However, no statistically significant difference was noted in their study.

In our study, we found that the diagnostic accuracy of the VNCa images was superior to that of the 70 keV images. This finding is consistent with recent studies conducted by Booz et al. [9] and Booz et al. [11], which demonstrated that color-coded VNCa images improved the diagnostic accuracy of evaluating disk herniation compared to standard gray-scale CT. It is likely that the high water content (ranging from 60 to 80%) within the nucleus pulposus [17] made the water-based images easier to identify bulging and protrusion.

In distinction to previous studies [8–11] that only focus one water-based image set, our study systematically compared the diagnostic performance of different water-based image sets with the standard gray-scale CT (70 keV image set). We found that, when evaluating IVDs, the VNCa image set demonstrated better diagnostic accuracy than both the 70 keV and water (iodine) image sets. Notably, regardless of the reconstruction algorithm used, the VNCa image set produced comparable results to the water (iodine) image set reconstructed by DLIR-H algorithm. These findings suggest that VNCa image are reliable for evaluating IVDs irrespective of reconstruction algorithms. However, further research is required to validate these findings.

The DLIR algorithm, which uses a dedicated deep neural network, has been proposed to reduce background noise and enhance image quality. In this study, we observed that DLIR-H helps to decrease background noise and enhance SNR and CNR in virtual monochromatic images (70 keV image set) when assessing IVDs. This finding aligns with our recent study comparing the DLIR algorithm to the ASiR-V algorithm for evaluating carotid atherosclerosis [14]. Additionally, we found that DLIR was beneficial for MD images as it decreased the SD of water-based MD images while maintaining similar WC values. These findings are consistent with studies conducted by Noda et al. [14] and Fukutomi et al. [18]. This indicates that DLIR-H helped reduce variability in WC values, providing more accurate and reproducible results with greater robustness. Besides, we found that images set reconstructed with DLIR demonstrated better interobserver agreement, which is of great value for reproducibility in clinical evaluation. To our knowledge, this is the first study investigating the combination of DLIR with water-based MD images for evaluating intervertebral discs. There is growing evidence suggesting that measuring water content in the intervertebral disks can help classify intervertebral disk degeneration [17, 19]. Further research is necessary to investigate the potential of using DLIR together with water-based MD images.

There are several limitations in this study. First, the retrospective single-center study design and relatively small sample size restrict the generalizability of our findings. Second, this study primarily focused on the display and morphological changes of IVDs, without evaluating spinal nerve root impingement, spinal cord compression, or the value of WC values in determining the stage of IVDs degeneration. Third, although efforts were made to minimize bias by partially removing annotations, the relatively short evaluation interval of 4 weeks between the three reconstructed image sets may have caused some recall bias. Fourth, it is not appropriate for patients suspected of having cervical spondylosis to undergo carotid DECTA. Additionally, the reference standard used for evaluating IVD in this study did not rely on MRI. In the future, it would be desired for the improvement of disc display by DLIR to be based on prospective cohort studies in patients with cervical spondylosis. Lastly, only a single-source rapid kV-switching dual-energy CT was used in this study. Therefore, further research with a larger sample size and different CT scanner vendors is needed to validate the findings of this study.

In conclusion, DLIR algorithm significantly improved the visualization of IVDs in the 70 keV and water (iodine) image sets, particularly for the water (iodine) image set. However, its improvement on color-coded VNCa image set was limited.

## Data Availability

The data used in current study were available on reasonable request.

## Abbreviation

CTA: Computed tomography angiography
IVD: Intervertebral discs
DECT: Dual-energy CT
VMI: Virtual monochromatic image
MD: Material decomposition
DLIR: Deep learning imaging reconstruction algorithm
VNCa: Virtual noncalcium
SNR: Signal-to-noise ratio
CNR: Contrast-to-noise ratio
ASiR-V: Adaptive statistical iterative reconstruction-Veo
WC: Water concentration
CV: Coefficient of variation
